# Linear phycoxyloglucan with oligo-xylose domains in the green seaweed *Ulva*: a unique hemicellulose

**DOI:** 10.1093/aob/mcag195

**Published:** 2026-07-17

**Authors:** Marie N Rapin, Lorna Murray, John H Bothwell, Ian H Sadler, Stephen C Fry

**Affiliations:** The Edinburgh Cell Wall Group, Institute of Molecular Plant Sciences, The University of Edinburgh, Daniel Rutherford Building, The King’s Buildings, Max Born Crescent, Edinburgh EH9 3BF, UK; EastChem School of Chemistry, The University of Edinburgh, The King’s Buildings, Edinburgh EH9 3JJ, UK; Department of Biosciences, Durham University, South Road, Durham DH1 3LE, UK; EastChem School of Chemistry, The University of Edinburgh, The King’s Buildings, Edinburgh EH9 3JJ, UK; The Edinburgh Cell Wall Group, Institute of Molecular Plant Sciences, The University of Edinburgh, Daniel Rutherford Building, The King’s Buildings, Max Born Crescent, Edinburgh EH9 3BF, UK

**Keywords:** Cellulose hydrogen-bonding, Chlorophyta, hemicellulose, nuclear magnetic resonance spectroscopy, oligo-glucan domain, oligosaccharide, oligo-xylan domain, phycoxyloglucan, sea lettuce, *Ulva linza* Linnaeus

## Abstract

**Background and Aims:**

The chlorophycean seaweed genus *Ulva* (sea lettuce and relatives) possesses cellulose and cell-wall-matrix polysaccharides operationally classified as ‘pectin-like’ (hot-water-extractable anionic ulvans and glucuronans) and hemicelluloses. Given that *Ulva* is only distantly related to streptophytes (land-plants plus charophytic algae), their wall polysaccharides are likely to diverge significantly. We therefore explored the little-known structure and properties of *Ulva* hemicellulose.

**Methods:**

Hemicellulose b was alkali-extracted from hot-oxalate-pretreated *Ulva linza* alcohol-insoluble residue, then digested with endo-β-xylanase, Driselase or endo-cellulase, yielding several oligosaccharides. These were separated by size on gel-permeation and preparative paper chromatography. Oligosaccharides were dissected by acid hydrolysis, glycosidase digestion and alkaline peeling; products were analysed by thin-layer chromatography. Purified oligosaccharides were also characterized by nuclear magnetic resonance (NMR) spectroscopy.

**Key Results:**

Thirteen di- to pentasaccharides were purified. On acid hydrolysis, they yielded only xylose (Xyl) and/or glucose (Glc). Each was digestible by either β-xylosidase or β-glucosidase, identifying the non-reducing terminus as β-Xyl or β-Glc, respectively. The rate of alkaline peeling, which attacks progressively from the reducing terminus, indicated (1→4) linkages; in each case, the smallest peeling product (i.e. originally the non-reducing terminus) was either Xyl or Glc, concurring with the glycosidase digestion result. Structures were deduced [β-(1→4)-linked Xyl-Xyl, Xyl-Xyl-Xyl, Xyl-Glc, Xyl-Xyl-Glc, Xyl-Xyl-Xyl-Glc, Glc-Glc, Glc-Xyl-Xyl, Glc-Glc-Xyl-Xyl and Xyl-Glc-Glc-Xyl-Xyl] and validated by NMR spectroscopy. Unexpectedly, NMR showed one oligosaccharide to be Glc-3-Glc-4-Glc, with the (1→3)-bond probably arising artefactually by trans-β-glucanase activity of the cellulase. *Ulva* hemicellulose hydrogen-bonded to cellulose more rapidly than any tested land-plant polysaccharide.

**Conclusions:**

The major hemicellulose of *Ulva* is a β-(1→4)-linked linear polysaccharide of Xyl and Glc, which we term phycoxyloglucan. The backbone has runs of up to three contiguous β-Xyl and three contiguous β-Glc residues, thus differing fundamentally from land-plant xyloglucan. Its structure confers high affinity for cellulose, suggesting potential biotechnological applications for this seaweed and contributing to the mechanical properties of the cell wall.

## INTRODUCTION

Chlorophytes are a taxonomic group comprising the vast majority of marine green algae (Fang *et al.*, 2017). They are distinct from the streptophytes, which are a monophyletic group containing the charophytic algae plus land plants. Chlorophytes are extremely diverse in size, morphology and environments. In this paper, we focus on a marine chlorophytic seaweed, *Ulva* (Fig. 1), which, like many chlorophytes, produces sulphated polysaccharides, possibly as an adaptation to life in highly ionic environments, such as seawater (Popper and Tuohy, 2010). Numerous species in this genus increasingly proliferate in eutrophicated coastal waters as harmful ‘green tides’. *Ulva* cell walls are remarkable because they contain both classic cellulose and anionic polysaccharides that are extractable in hot, mildly acidic water; namely glucuronan and a class of unique polymers known as ulvans (branched sulphated polysaccharides rich in uronic acids and rhamnose) (Lahaye and Robic, 2007). Because glucuronan and ulvan can be extracted in hot, mildly acidic water, as for land-plant pectin, we refer to them as ‘pectin-like’. Indeed, ulvan has an alternating backbone somewhat reminiscent of a land-plant pectic domain (rhamnogalacturonan-I) and a charophyte fucoglucuronan (Tan *et al.*, 2026). The ability of ulvans to form hydrogels, in addition to their multiple bioactive properties, makes them interesting for biomedical applications.

**Fig. 1.**
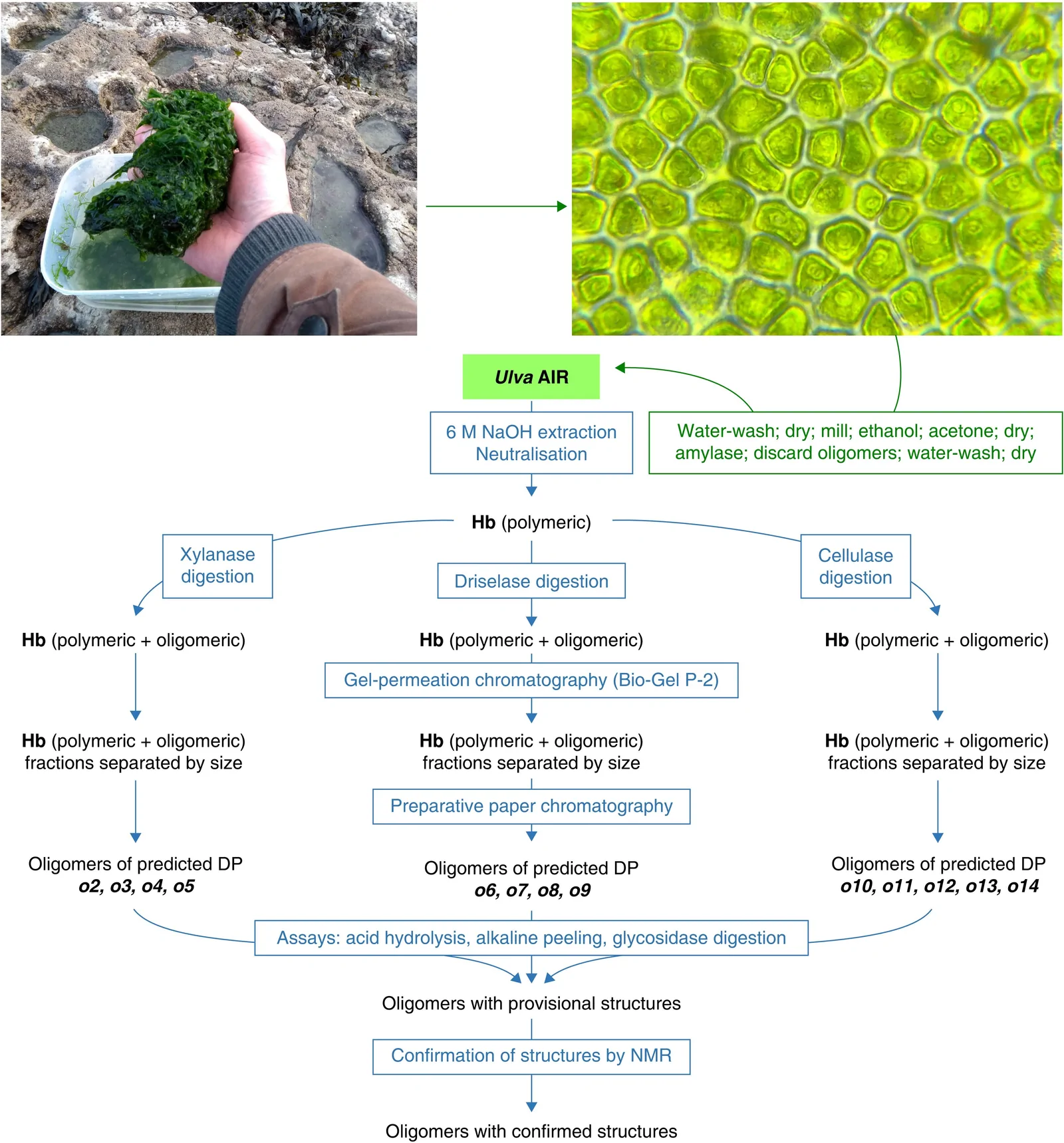
Overall scheme of production and analysis of xylanase, Driselase and cellulase digestion products. The images at the top show collection of the green seaweed *Ulva* cf. *linza* (ribbon-type biomass) and a bright-field image looking down onto the upper layer of cells in the algal thallus. Thalli in this species are bilayers, and each cell is ∼5–10 µm across. Abbreviations: **Hb**, hemicellulose b; ***o2*–*o14***, oligosaccharides obtained.

However, in addition to the relatively well-studied cellulose and pectin-like polysaccharides, *Ulva* cell walls also possess an alkali-extractable fraction that is tightly bound to the cellulose. In land-plants, such a fraction would be termed ‘hemicellulose’. Land-plant hemicelluloses are commonly used for the production of biofuels, and in packaging, food production and drug-delivery systems, thanks to their thickening, emulsifying, stabilizing and gelling properties. Hemicelluloses from algae, in contrast, have mostly been of academic interest in elucidating the evolutionary history of cell walls. *Ulva* hemicellulose is a xylose- and glucose-rich polymer (Ray and Lahaye, 1995; Lahaye and Robic, 2007) but, to our knowledge, it remains poorly characterized (Lahaye *et al.*, 1994; Chattopadhyay *et al.*, 2007).

In this paper, we clarify its structure and propose that *Ulva* contains a phycoxyloglucan with oligo-xylose domains, which serves, among other functions, to reinforce the algal structure by its affinity to cellulose.

## MATERIALS AND METHODS

### Seaweed

*Ulva* sp. (provisionally identified as *Ulva linza*) was wild-collected at North Berwick, UK.

### Enzymes and chemical supplies

Xylanase (E-XYRU6; from rumen microorganisms), cellulase (E-CELBA; from *Bacillus amyloliquefaciens*), β-glucosidase (E-BGLUC; from *Aspergillus* sp.) and β-xylosidase (E-BXSR-1KU; from *Selenomonas ruminantium*) were sourced from Megazyme (Auchincruive, UK); Driselase (D8037, from a basidiomycete) was from Sigma-Aldrich (Poole, UK). Xylopentaose, cellopentaose and most polysaccharides were from Megazyme; other chemicals and solvents were from Sigma-Aldrich. Xylobiose was prepared in our laboratory by digestion of xylan with Driselase and purified by preparative paper chromatography. The land-plant polysaccharides used (for preview, see Fig. 2) were as follows: wheat flour arabinoxylan (medium viscosity; P-WAXYM; Megazyme), tamarind seed xyloglucan (a generous gift from Mr K. Yamatoya, Dainippon Pharmaceutical Co., Osaka, Japan), citrus homogalacturonan (‘polygalacturonic acid, sodium salt’, ∼250 kDa; P3850; Sigma), barley mixed-linkage glucan (β-glucan, medium viscosity; P-BGBM; Megazyme) and beechwood 4-*O*-methylglucuronoxylan (M5144; Sigma). Gel-permeation chromatography materials were from Bio-Rad (Kidlington, UK). All water used was deionized. Silica-gel thin-layer chromatography (TLC) plates (plastic and aluminium-backed) were from Merck (Glasgow, UK). Activated charcoal columns (graphitized non-porous carbon, Supelco, Supelclean™ ENVI-Carb™ SPE Tube, volume 1 mL) were also from Merck.

**Fig. 2.**
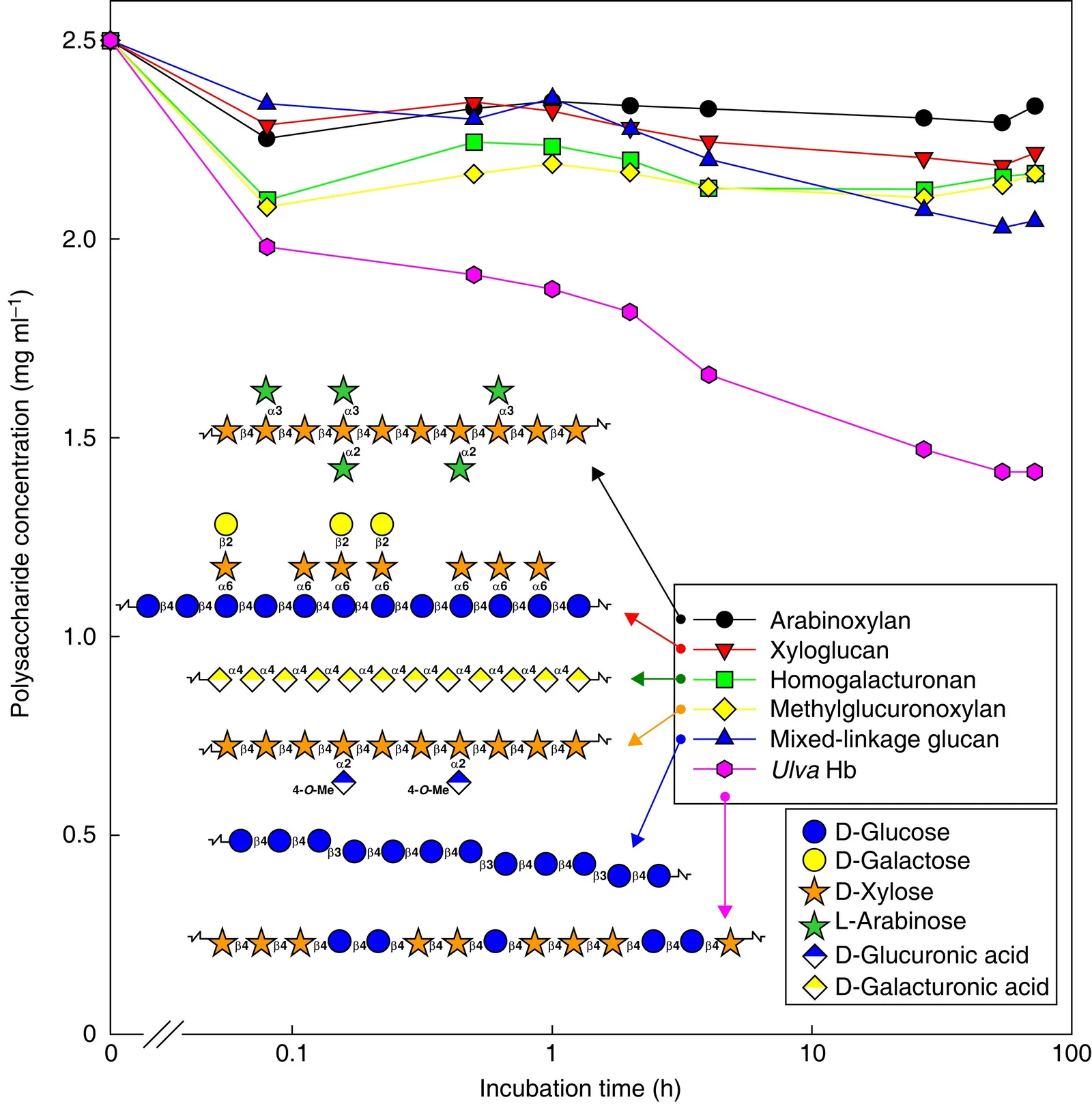
Structures of polysaccharides used in this paper, and the results of an experiment (described at the end of the Results section) on their ability to hydrogen-bond to cellulose. For the experiment, polysaccharide solutions (2 mL; initial concentration 2.5 mg mL^−1^ in water) were incubated with Whatman No. 1 paper strips (125 mg) for various durations at 20 °C on a rotating wheel. At each time point, a 5 µL aliquot of solution was removed for carbohydrate assay. For each polymer solution, a dilution series was prepared, and 2 µL was dot-blotted onto a silica-gel TLC plate and stained with thymol. The intensity of each dot was measured and used to create a reference curve, to which a logarithmic function was fitted. All samples were dot-blotted and stained on the same TLC plate. The intensity of each dot was measured and the corresponding sugar concentration calculated. Each experiment was run in triplicate. The ‘time 0’ sample was taken before the addition of cellulose. Partial structures of the polysaccharides are illustrated, using the sugar symbols shown at bottom right. The structure of the *Ulva* ‘phycoxyloglucan’ Hb is based on the present paper.

### Polysaccharide extraction

Fresh algal biomass was thoroughly washed and freeze-dried. The dried material was hand-milled by mortar and pestle. Alcohol-insoluble residue (AIR) was prepared by rinsing several times with, successively, 75 % ethanol, 96 % ethanol and acetone.

Algal AIR (0.5–5.0 mg mL^−1^) was de-starched by stirring with 50 % (v/v) human saliva (Olaitan and Northcote, 1962) in 20 mM lutidine (acetate^−^, pH 6.5 containing 0.25 % chlorobutanol), at 37 °C for 16 h. The reaction was stopped by the addition of formic acid to 20 % v/v. The hydrolysate was centrifuged, and the supernatant, containing starch oligomers, was discarded. The pellet, containing non-starch polysaccharides, was washed with water, dried, re-suspended in water, re-dried and, finally, made up to the desired concentration.

Ulvan- and glucuronan-rich polysaccharide (‘pectin-like’) fractions were prepared by incubation of AIR successively in 0.2 M ammonium oxalate (pH 4.1) at 100 °C for 2 and 16 h (extracts P1 and P2, respectively). Hemicellulose was obtained by incubation of the remaining insoluble residue in 6 M NaOH at 37 °C for 72 h. The pH of the hemicellulosic fraction was then adjusted to ∼4.2 with acetic acid, and the fraction was dialysed against water. The resulting soluble fraction (Hb) was separated by centrifugation from the newly formed precipitate (Ha). The remaining insoluble algal material was washed in 0.2 M acetate (Na^+^, pH 4.7) to obtain fraction W, in addition to the final insoluble fraction, α-cellulose (αC).

### Acid hydrolysis

Polysaccharidic material (5 mg) was hydrolysed with 1 mL of 2 M trifluoroacetic acid at 120 °C for 1 h. The hydrolysate was dried, re-dissolved in water, re-dried and, finally, made up to the desired concentration.

### Alkaline peeling

Samples were incubated in 0.2 M NaOH for 0, 6, 60 and 125 min at 50 °C, then neutralized with acetic acid (Franková and Fry, 2012).

### Enzymic hydrolysis

Solubilized algal polymers and oligomers (0.2–0.3 %, w/v) were digested with β-xylanase [final concentration 2.7 U mL^−1^ in 167 mM acetate (pyridinium^+^) buffer, pH 4.7, for 72 h at 20 °C], Driselase (final concentration 0.05 % w/v in the same buffer for 72 h at 37 °C) or cellulase (final concentration 3.3 U mL^−1^ in the same buffer for 240 h at 37 °C).

Algal oligomers (0.2 % w/v) were treated with β-glucosidase (final concentration 0.16 U mL^−1^, in the same buffer for 1 h at 50 °C) or β-xylosidase [final concentration 27 U mL^−1^, in 25 mM collidine (acetate^−^, pH 6.0), for 16 h at 37 °C].

### Chromatography

Each preparative sample (∼50 mg, in 3 mL water) was separated by gel-permeation chromatography on a ∼120 mL column of Bio-Gel P-2 at room temperature (∼20 °C) with pyridine/acetic acid/water (PyAW; 1:1:98) as eluant (∼30 mL h^−1^). At least fifty 3 mL fractions were collected, i.e. 150 mL recovered. Eluates were assayed for carbohydrate by dot-blotting of 5 µL portions on a silica-gel plate and staining with thymol (see below).

Paper chromatography was performed on Whatman No. 1, usually in butan-1-ol/acetic acid/water (BAW; 12:3:5, v/v/v) by the descending method for 16 h. Markers and a transect of the unknown sugar sample were stained with aniline hydrogen-phthalate (Fry, 2000). For preparative samples, selected unstained portions were cut off the sheet and extracted with 75 % ethanol by centrifugation (Eshdat and Mirelman, 1972).

Silica-gel TLC plates were developed in ethyl acetate/pyridine/acetic acid/water (EPAW, 6:3:1:1) or BAW (2:1:1 or 4:1:1) or butan-1-ol/ethanol/water (BEW, 3:1:1) or ethyl acetate/pyridine/propan-1-ol/acetic acid/water (EPPAW, 5:2:2:1:1). Separated sugars were stained with thymol/H_2_SO_4_ (Jork *et al.*, 1994).

### Nuclear magnetic resonance spectra

Samples were purified further on 1-mL activated charcoal columns; this removed unidentified impurities stemming from the binding agent in the TLC plates, which otherwise interfered with the nuclear magnetic resonance (NMR) spectra. Columns were conditioned with 3 mL pyridine/acetic acid/water (PyAW; 1:1:98), and samples were loaded (0.5 mL at 5 mg mL^−1^, in the same buffer). Columns were washed with the same buffer, then samples were eluted with, sequentially, 10, 20 and 30 % (v/v) acetonitrile in water (each 2 × 1.5 mL). Fractions with the highest sugar contents (measured by dot-blotting, as above) were combined and dried, then examined by NMR spectroscopy.

One- and two-dimensional proton and carbon-13 NMR spectra were recorded on a Bruker AVANCE NEO instrument (18.8 T; 800 MHz for protons) with *d*_4_-methanol as solvent. Proton spectra were referenced to the residual CD_2_HOD signal at 3.33 ppm, and carbon-13 spectra were referenced to CD_3_OD at 49.0 ppm. Chemical shifts are given in parts per million (δ) relative to tetramethylsilane, and scalar coupling constants (*J*) are given in hertz. These data were obtained by detailed analysis of one-dimensional proton and carbon-13 spectra, selective one-dimensional proton correlation spectra (TOCSY) propagated from H-1 signals, two-dimensional proton correlation spectra (COSY), and two-dimensional proton–carbon one-bond (HSQC) and multiple-bond (HMBC) correlation spectra carried out on every sample.

## RESULTS

### Analysis of extracts from *Ulva*

After being de-starched with α-amylase, *Ulva* AIR was submitted to sequential extraction. We confirmed the previous report (Rapin *et al.*, 2023) that hot oxalate-extracted fractions (P1 and P2) made up about half the total extracted weight (i.e. sum of P1, P2, Ha, Hb, wash and α-cellulose) and were composed of sugar residues typical of ulvan and glucuronan: glucuronic acid and rhamnose, together with smaller amounts of xylose and glucose (Fig. 3). The alkali-soluble (hemicellulosic) extracts were markedly different, featuring mostly glucose and xylose residues in approximately equal quantities. The neutral wash (W) after alkaline extraction resembled the hemicellulose extracts. The final, inextractable fraction (αC) consisted mainly of glucose residues, as expected for cellulose, plus ≤20 % xylose [which, in land-plants, often remains trapped within cellulose preparations, possibly because of difficulties in disrupting hemicellulose–cellulose interactions (Wang *et al.*, 2022)].

**Fig. 3.**
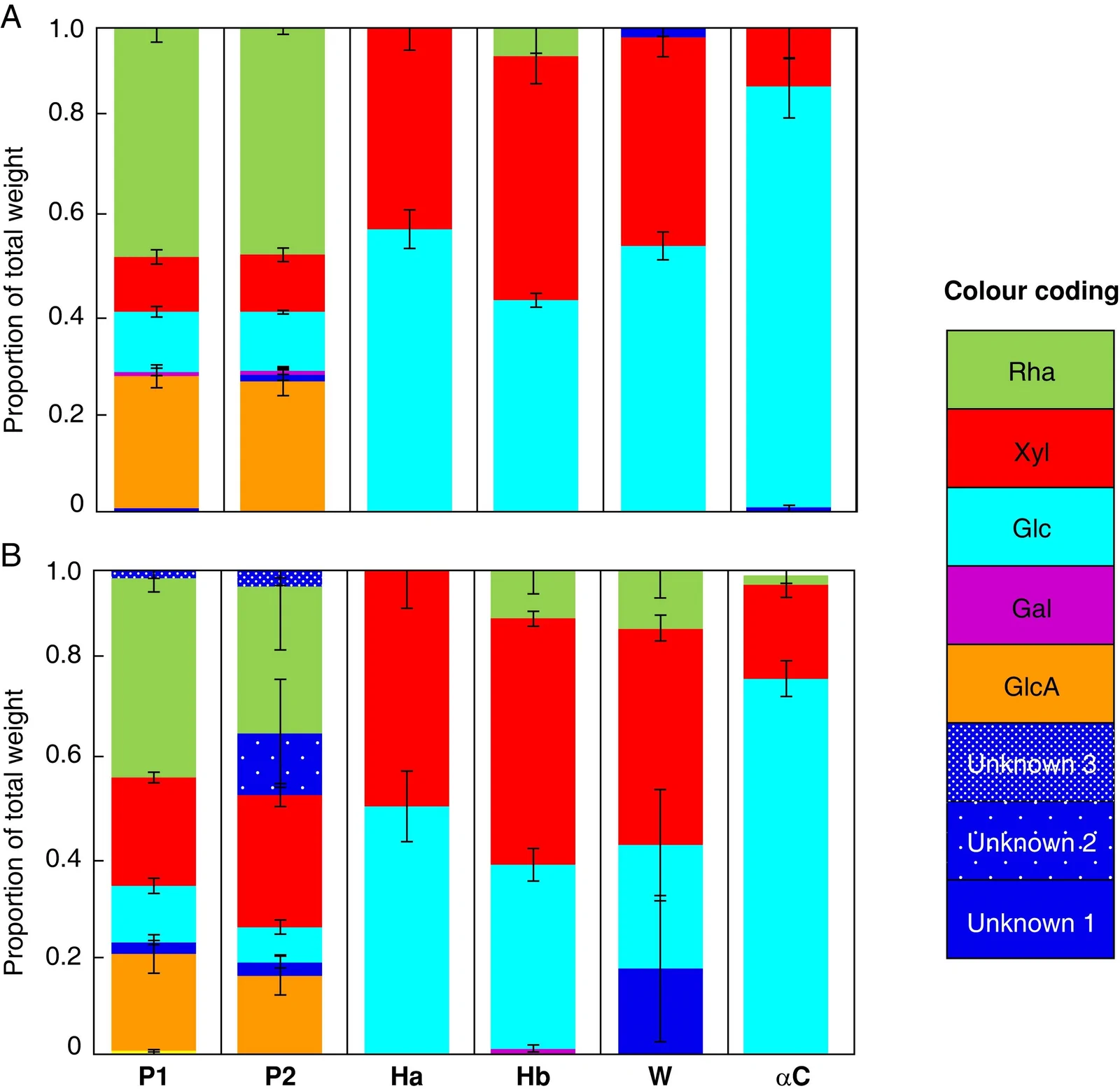
Relative quantities of sugar residues in six polysaccharide fractions extracted from *Ulva* cell walls. Chromatography solvents (always with two ascents) were: (A) BAW 4:1:1; or (B) EPAW 6:3:1:1. Other details are as described by Rapin *et al.* (2023). Polymer fractions, extracted from amylase-pretreated *Ulva* AIR, were as follows: P1 and P2, ‘pectin-like’ polysaccharides; Ha and Hb, hemicelluloses a and b; W, mildly acidic cold-water wash after alkaline extraction; αC, residual ‘α-cellulose’. Three aliquots of each AIR preparation were extracted to produce the listed polymer fractions, then three aliquots of each fraction were separately acid-hydrolysed and analysed by TLC as described before (Rapin *et al.*, 2023, 2024). Error bars are the s.d., with *n* = 3 (relating to the number of analysed polysaccharide fractions from the same AIR sample).

### Specificity of enzymes used

We used xylanase, Driselase and cellulase to dissect *Ulva* hemicellulose b enzymically to oligosaccharides, facilitating further structural characterization. Initially, we tested the specificity of these three enzyme preparations by incubating them individually with authentic β-Glc- and β-Xyl-based oligosaccharides (Fig. 4A–C). The undigested substrates are illustrated in Fig. 4D. [Note that, throughout this paper, by ‘-osidase’ and ‘-anase’ we imply exo-hydrolase and endo-hydrolase activity, respectively.]

**Fig. 4.**
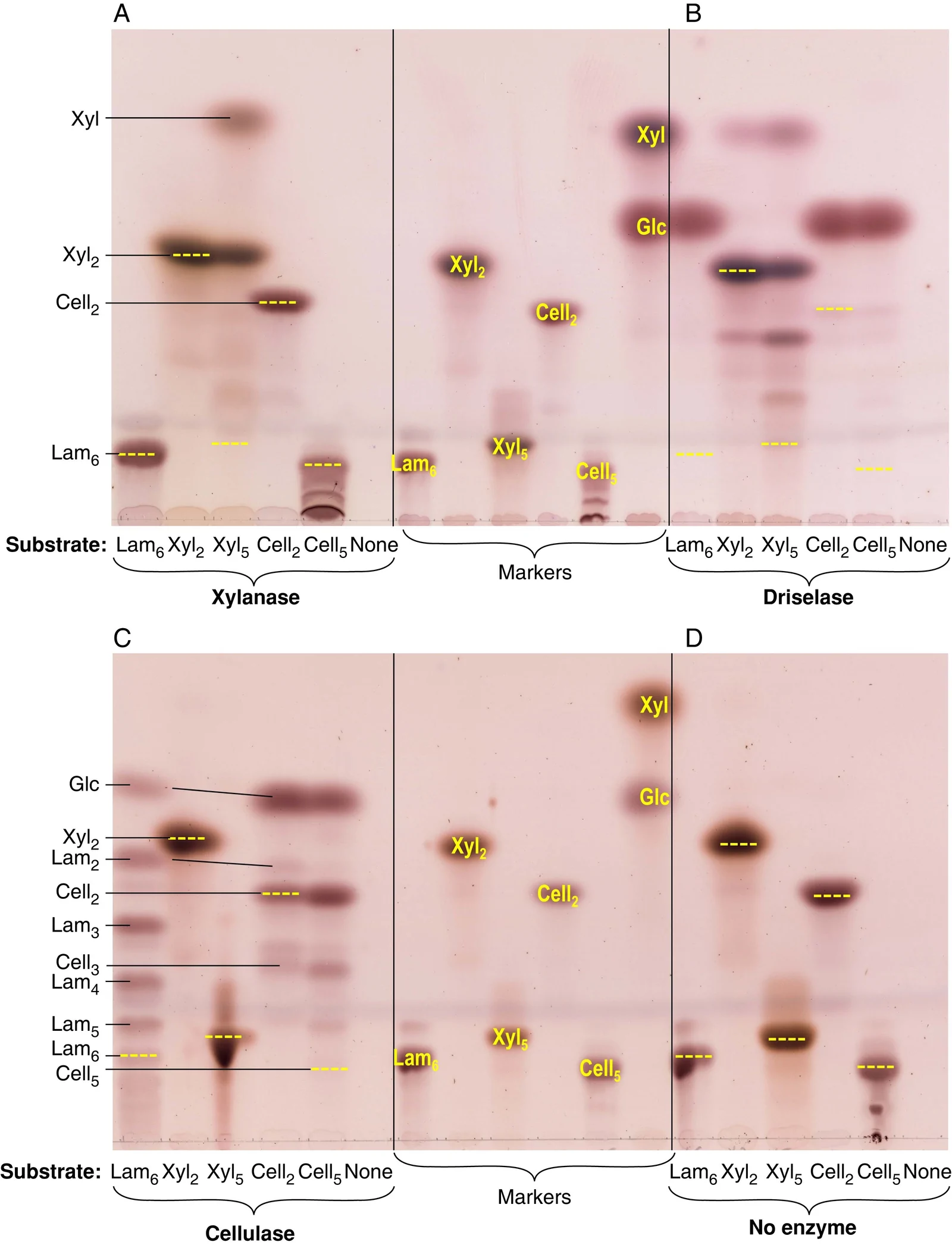
Enzymic digestion of authentic oligosaccharides. The actions of xylanase (A), Driselase (B), cellulase (C) and plain buffer (D) were tested on a range of commercial substrates (Cell_2_, cellobiose; Cell_5_, cellopentaose; Lam_6_, laminarihexaose; Xyl_2_, xylobiose; Xyl_5_, xylopentaose). Substrate-free controls (‘None’) were also performed for each enzyme. Dashed yellow lines indicate positions of the starting materials. Products were analysed by TLC in BAW 4:1:1 with two ascents.

The xylanase is capable of hydrolysing land-plant xylans. It was inactive on xylobiose and on the β-Glc-homo-oligosaccharides tested, but digested xylopentaose to xylobiose + Xyl (Fig. 4A). Thus, the xylanase was able to cleave only xylosyl linkages, not glucosyl. We cannot tell exactly how many Xyl residues needed to be located towards the non-reducing end of the cleaved bond, only that at least the adjacent one needed to be Xyl. Therefore, any oligosaccharide generated from the hemicellulose by the xylanase must have possessed at least one additional Xyl residue towards the non-reducing end. For example, if a polysaccharide yielded XGGXX, the original polysaccharide can be deduced to have contained the sequence XXGGXX [where X stands for Xyl, G for Glc, and all sugars are β-(1→4)-linked].

Driselase (Fig. 4B) digested all the Glc-based homo-oligosaccharides [both (1→3)- and (1→4)-linked], very largely to the monosaccharide Glc, with only traces of disaccharides as by-products. It left xylobiose largely intact, but accompanied by small quantities of Xyl plus a trisaccharide, suggesting a minor trans-xylosidase reaction (2 Xyl_2_ ↔ Xyl_3_ + Xyl). Driselase hydrolysed xylopentaose largely to xylobiose + Xyl, but again accompanied by a trace of tri- and tetrasaccharides, suggesting transxylosylation. This shows that Driselase contains β-glucanase, β-glucosidase and xylanase activities, and probably also trans-β-xylosidase, but not β-xylosidase.

The cellulase, finally, was inactive on both the tested Xyl homo-oligosaccharides (Xyl_2_ and Xyl_5_; Fig. 4C). In the incubation time allowed, it was able to split cellobiose partly into Glc, indicating the presence of β-glucosidase activity. During this process, it produced small amounts of a different disaccharide, in addition to at least two tri- and tetrasaccharides, indicating transglycosylation. The minor disaccharide approximately co-migrated with laminaribiose [the β-(1→3)-dimer; Fig. 4C, left-hand lane]. Cellopentaose gave Glc, cellobiose and small amounts of tri- and tetrasaccharides. Authentic laminarihexaose was digested to Glc plus a ladder of presumably mainly β-(1→3)-linked oligosaccharides, showing that the ‘cellulase’ was not specific for β-(1→4)-bonds; furthermore, laminarihexaose gave a minor disaccharide that co-migrated with cellobiose. Thus, the cellulase was able to interconvert β-(1→3)- and β-(1→4)-linkages by transglycosylation reactions such as:

2 **G4G** ↔ G3G4G + G followed by endo-hydrolysis (G3G4G → **G3G** + G) and

2 **G3G** ↔ G4G3G + G followed by endo-hydrolysis (G4G3G → **G4G** + G).

The cellulase was able to cleave only glucosyl linkages, not xylosyl. However, we cannot tell exactly how many Glc residues needed to be located towards the non-reducing end of the cleaved bond, only that at least the first one needed to be Glc. Therefore, any oligosaccharide generated from the hemicellulose by the cellulase must have possessed at least one additional Glc residue towards the non-reducing end. For example, if a polysaccharide yielded XXXG, the original polysaccharide can be deduced to have contained the sequence GXXXG.

### Enzymic digestion of *Ulva* polysaccharide fractions

*Ulva* polymer fractions P1, P2, Hb and αC were treated with the above xylanase preparation (Fig. 5A); the low yield of Ha precluded further analysis. The only solubilized polysaccharide fraction to show detectable hydrolysis products was Hb, which produced three oligomers, labelled X1, X2 and X3. This was surprising, because Xyl–Xyl linkages are not expected in *Ulva* polysaccharides (Chattopadhyay *et al.*, 2007). However, total acid hydrolysis showed that X1, X2 and X3 were composed of both Glc and Xyl residues, hence the enzyme was clearly able to digest polymers containing both these residues, not only Xyl.

**Fig. 5.**
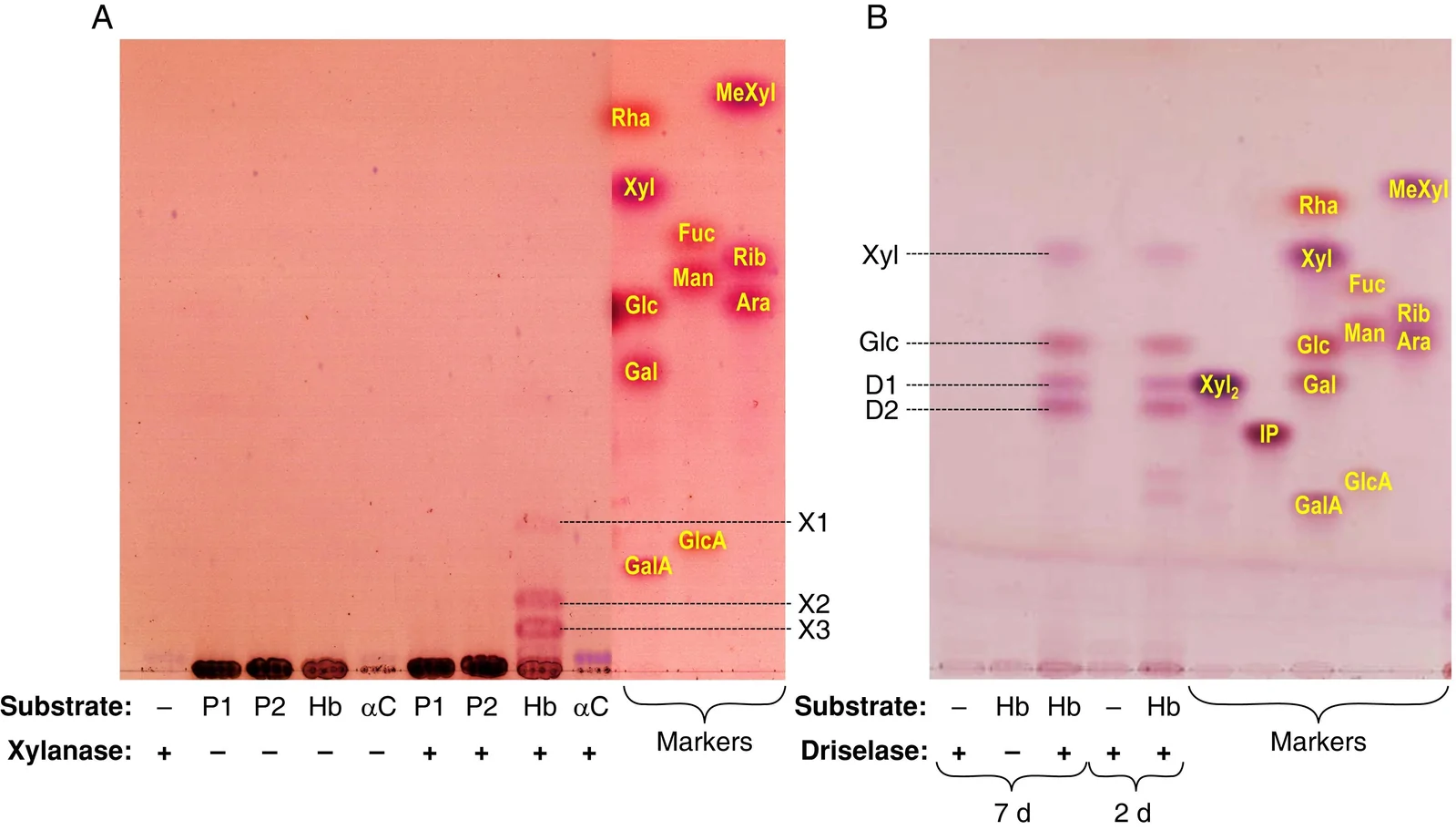
Digestion of selected *Ulva* polysaccharide fractions by xylanase and Driselase. (A) *Ulva* pectin-like polysaccharides (P1, P2), hemicellulose b (Hb) and α-cellulose (αC) were incubated with and without xylanase. Xylanase digestion products of Hb are labelled X1, X2 and X3. (B) *Ulva* hemicellulose b was incubated with and without Driselase for 2 or 7 days. Driselase digestion products are labelled Xyl, Glc, D1 and D2. TLC solvent was in BAW, 4:1:1, with two ascents. In A, the TLC was scanned in two parts, which have been joined digitally.

Further samples of fractions P2 and Hb were treated with Driselase, a mixture of enzymes including cellulase, pectinases and xylanase (Fry, 2000), which is active on β-1,4 oligo-xylans, β-1,4 oligo-glucans and β-1,3 oligo-glucans, as described above. The *Ulva* ‘pectin’ P2 fraction did not release any detectable oligomer; however, Hb released four main products: the monosaccharides Xyl and Glc plus two unknowns (D1 and D2) (Fig. 5B). As expected, the monosaccharides did not change upon treatment with hot acid. D1 co-migrated with xylobiose and stained in an identical fashion, but upon acid hydrolysis it released both Xyl and Glc. D2 migrated more slowly on TLC, and was thus probably a larger oligosaccharide; it also comprised Xyl and Glc residues.

### Oligomer purification

The above experiments suggested that *Ulva* Hb contains both glucose-rich and xylose-rich sections, most likely β-1,4-linked. To explore this in more depth, we treated *Ulva* Hb with xylanase, Driselase and cellulase and analysed the resulting oligomers. Cellulase was included because it targets β-1,4-glucans and was thus expected to generate intact short xylose-rich sections. Fig. 1 (methodology) and Table 1 (summary of data) will help the reader navigate the manuscript.

**Table 1.** Summary of properties of oligosaccharides obtained by enzymic digestion of *Ulva* hemicellulose b

Oligosaccharide	Obtained via	DP by GPC	Monomers after acid hydrolysis (Supplementary Data Fig. S3)	*R*_Glc_ on PC^‡^	Reducing terminus by staining^¶^	Co-migrates on TLC with	Main products of β-xylosidase	Main products of β-glucosidase	Non-reducing terminus^§^	Deduced identity of oligosaccharide^†^	Extrapolated structure within hemicellulose
**o2**	Xylanase	5	Xyl > Glc	0.11	Xyl		Xyl + unknowns	Unknowns	Xyl	★β4⬤β4⬤β4★β4★	★β4★β4⬤β4⬤β4★β4★
**o3**	Xylanase	4	Xyl ≈ Glc	0.19	Xyl		Some unknowns	Glc, Xyl-Xyl	Glc	⬤β4⬤β4★β4★	★β4⬤β4⬤β4★β4★
**o4**	Xylanase	3	Xyl, Glc	0.41	Xyl		Some unknowns	Glc, Xyl-Xyl	Glc	⬤β4★β4★	★β4⬤β4★β4★
**o5**	Xylanase	2	Xyl	0.78	Xyl	Xylobiose	Xyl	–	Xyl	★β4★	★β4★β4
**o6**	Driselase	3	Xyl	0.39	Glc		Xyl	–	Xyl	★β4★β4★	★β4★β4★
**o7**	Driselase	3	Xyl > Glc	0.40	Xyl		Xyl > Glc	–	Xyl	★β4★β4⬤	★β4★β4⬤
**o8**	Driselase	2	Xyl ≈ Glc	0.67	Glc		Xyl ≈ Glc	–	Xyl	★β4⬤	★β4⬤
**o9**	Driselase	2	Xyl	0.76	Xyl	Xylobiose	Xyl	–	Xyl	★β4★	★β4★
**o10**	Cellulase	4	Xyl > Glc^F^	0.18	Glc		Xyl > Glc	–	Xyl	★β4★β4★β4⬤	⬤β4★β4★β4★β4⬤
**o11**	Cellulase	3	Glc >> Xyl	0.37	Glc		–	Glc	Glc	⬤β3⬤β4⬤	⬤β4⬤β3⬤β4⬤
**o12**	Cellulase	3	Xyl > Glc	0.37	Glc		Xyl > Glc	–	Glc	★β4★β4⬤	⬤β4★β4★β4⬤
**o13**	Cellulase	2	Glc	0.58	Glc	Cellobiose	–	Glc	Glc	⬤β4⬤	⬤β4⬤β4⬤
**o14**	Cellulase	2	Xyl ≈ Glc^F^	0.67	?		Xyl ≈ Glc	–	Xyl	★β4⬤	★β4⬤

^†^⬤, glucose; ★, xylose. All deduced identities were confirmed by NMR spectroscopy (Table 2; Supplementary Data Table S1).

^‡^Paper-chromatographic mobility (in BAW, 12:3:5 for 72 h) relative to that of glucose.

^¶^Pentose (Xyl) or hexose (Glc) as judged by colour of staining with aniline hydrogen-phthalate (reddish indicating pentose; brown indicating hexose).

^§^As judged by monosaccharide produced by alkaline peeling.

^F^Plus a trace of an unidentified monosaccharide running slightly faster than fucose.

The products formed by xylanase, Driselase and cellulase were size-fractionated by gel-permeation chromatography on Bio-Gel P-2 (Supplementary Data Fig. S1A–C). Each enzyme gave an undigested fraction [in the void volume, i.e. degree of polymerization (DP) greater than ∼12], a broad peak containing oligomers, and a shoulder containing monosaccharides. Aliquots from selected fractions were analysed by TLC, each enzyme giving a unique ‘ladder’ (Fig. 6). On the basis of the TLC results, selected Bio-Gel fractions were fractionated further by preparative paper chromatography (Supplementary Data Fig. S1D–F), resulting in three series of oligomers, labelled **o2–o5, o6–o9** and **o10–o14** from xylanase, Driselase and cellulase, respectively. Some oligomers co-migrated on paper chromatography and were re-separated via preparative TLC, such as compounds **o6** and **o7** and compounds **o11** and **o12** (Supplementary Data Fig. S2).

**Fig. 6.**
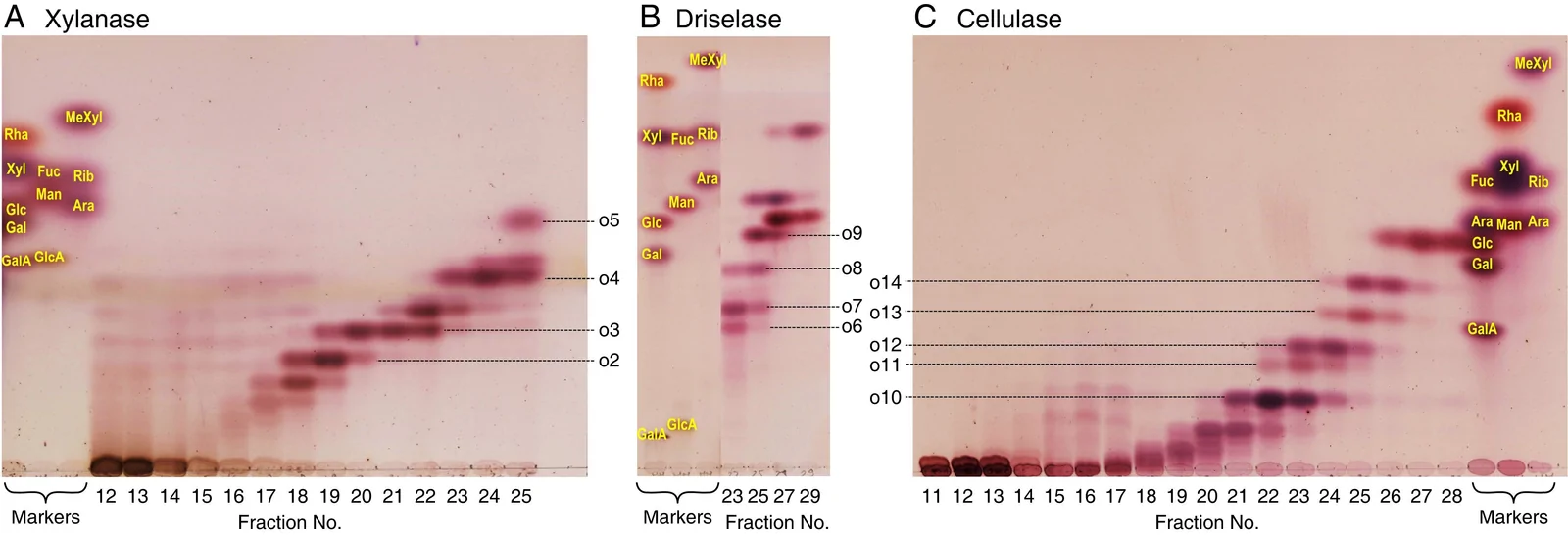
Oligosaccharide ladders produced by enzymic digestion of *Ulva* hemicellulose. *Ulva* hemicellulose b was digested with xylanase (A), Driselase (B) or cellulase (C), and the products were size-fractionated by gel-permeation column chromatography (see Supplementary Data Fig. S1A–C). Aliquots of the column fractions were analysed by TLC in BAW, 2:1:1, with one ascent (A), EPAW, with two ascents (B), and BAW, 4:1:1, with two ascents (C). In B, two parts of the same TLC were scanned and have been joined digitally.

### Estimation of oligosaccharide DP

We used the elution behaviour of oligosaccharides on gel-permeation chromatography to approximate their degrees of polymerization, assuming that all oligosaccharides behaved in a similar manner regardless of their sugar compositions (Supplementary Data Fig. S1A–C); these estimations were confirmed by the following TLC assays and NMR data. Oligomers **o5**, **o8**, **o9**, **o13** and **o14** were dimers; **o4**, **o6**, **o7**, **o11** and **o12** were trimers; **o3** and **o10** were tetramers; and **o2** was a pentamer.

### Monomeric composition of algal oligomers

Oligomers **o2** to **o14** were fully hydrolysed and the products separated by TLC (Supplementary Data Fig. S3). They were all composed very predominantly of Xyl and/or Glc in varying ratios, as summarized in Table 1.

### Co-migration assays

Compounds **o5** and **o9**, generated by xylanase and Driselase, respectively, both co-migrated with 1,4-β-xylobiose on TLC in two different solvent systems (Fig. 7B, C; Supplementary Data Fig. S4A). Coupled with their estimated DPs and monomer composition, **o5** and **o9** are both concluded to be 1,4-β-xylobiose, which we have shown to be resistant to xylanase and Driselase (Fig. 4).

**Fig. 7.**
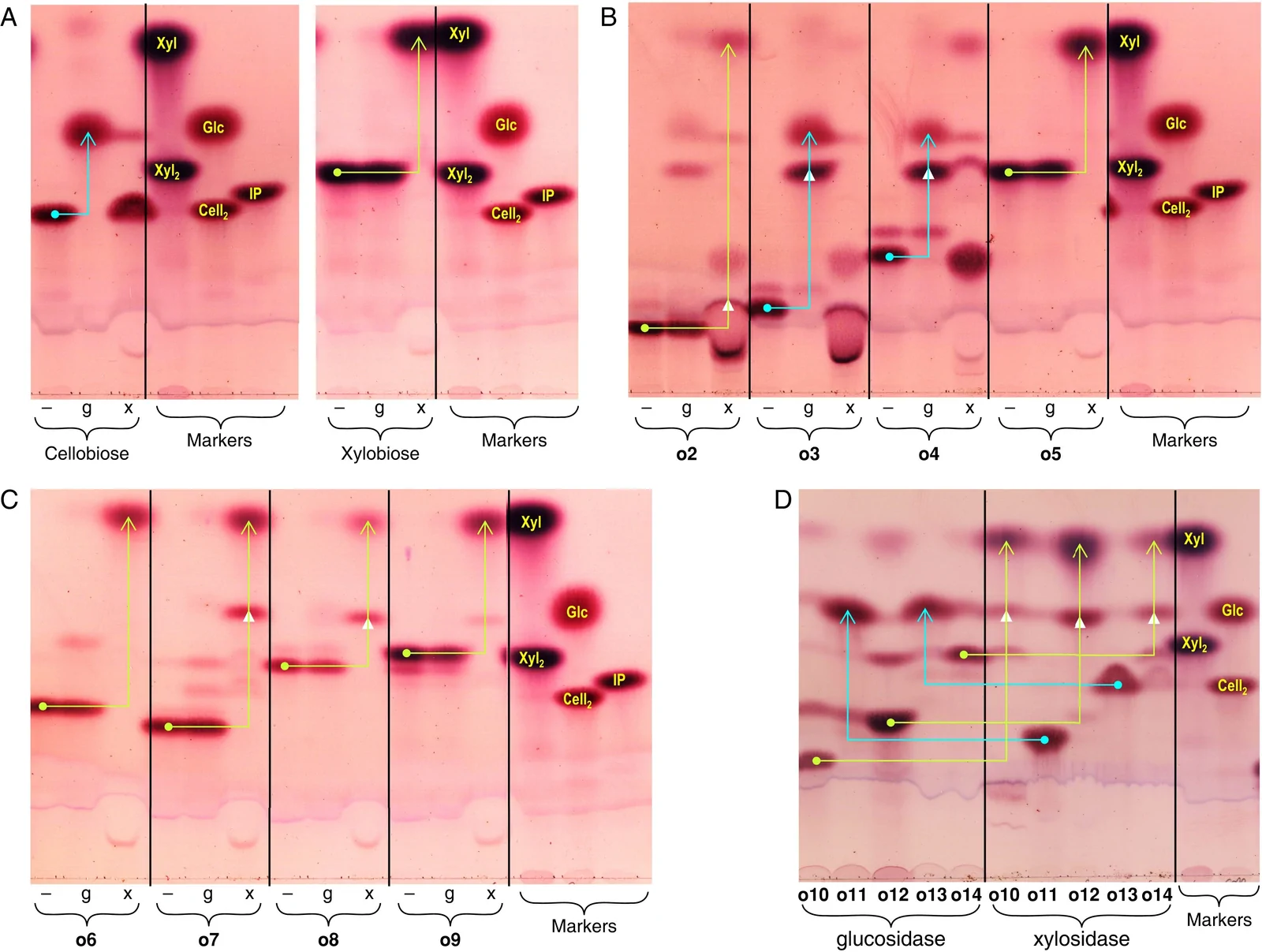
Glycosidase action on oligosaccharides from *Ulva* hemicellulose b. Each oligosaccharide was incubated in plain buffer (−) or with β-glucosidase (g) or β-xylosidase (x). (A) The substrates were authentic xylobiose (Xyl_2_) and cellobiose (Cell_2_), demonstrating the specificity of the enzymes. (B–D) *Ulva* oligosaccharides **o2** to **o14** generated by xylanase (B), Driselase (C) or cellulase (D). In D, plain buffer samples were omitted, but in each case the enzyme that did not digest the oligosaccharide effectively serves this role. The products were separated by TLC in BAW 2:1:1 with one ascent. The arrows indicate the conversion of starting material (tail of arrow; filled circle) to the monosaccharide that was the original non-reducing terminus of the oligosaccharide (arrowhead). Cyan arrows, glucosidase digestion; yellow arrows, xylosidase digestion. The solid white arrowhead (if any) indicates the remainder of the oligosaccharide (still bearing the original reducing terminus) after glycosidase digestion. Abbreviation: IP, isoprimeverose.

Compound **o13** co-migrated with cellobiose in two different solvent systems (Fig. 7D; Supplementary Data Fig. S4B) and yielded only Glc on acid hydrolysis; it is thus proposed to be cellobiose, which is indeed only partly hydrolysed by the cellulase in the conditions used (Fig. 4C).

### Using (exo-) glycosidases to explore the non-reducing termini

#### Authentic oligosaccharides

To investigate the sugar residues at the non-reducing termini of the oligosaccharides, we treated all 13 of these with, separately, β-glucosidase and β-xylosidase (Fig. 7). Authentic xylobiose and cellobiose, tested as controls, were hydrolysed essentially only by the expected enzyme, demonstrating the effectiveness and specificity of the two enzymes (Fig. 7A). The xylosidase preparation had a very slight ability to hydrolyse cellobiose.

#### Xylanase-generated oligosaccharides

Each of the xylanase-generated oligosaccharides, **o2–o5**, initially gave a single main band, with only minor contaminants (Fig. 7B). The disaccharide **o5** was hydrolysed by xylosidase to xylose but unaffected by glucosidase, supporting its identity as xylobiose. The trisaccharide (**o4**) was completely hydrolysed by glucosidase to Glc plus xylobiose, suggesting that it was Glc-Xyl-Xyl. Supporting this, **o4** was largely unaffected by xylosidase. The minor band in **o4** was unaffected by glucosidase but was hydrolysed by xylosidase, releasing Xyl; it might have been Xyl-Glc-Xyl. Glucosidase hydrolysed the tetrasaccharide **o3** to Glc plus xylobiose (Fig. 7B), indicating that it was Glc-Glc-Xyl-Xyl. It was unaffected by xylosidase. The pentasaccharide **o2** was more difficult to characterize. Xylosidase released Xyl plus an apparent trisaccharide, possibly indicating Xyl-Xyl-Glc-US-US (where US is an unspecified sugar) or a branched pentasaccharide with two Xyl non-reducing termini. However, glucosidase also acted on a minor component of **o2**, releasing Glc plus xylobiose, perhaps suggesting that Glc-Glc-Glc-Xyl-Xyl was also present as a minor component. Thus spot ‘**o2**’ was a mixture of oligosaccharides possessing Xyl and Glc non-reducing termini.

#### Driselase-generated oligosaccharides

Each Driselase-generated oligosaccharide, **o6–o9**, was initially a single main band, although **o8** and **o9** showed slight mutual cross-contamination (Fig. 7C). The trisaccharide **o6** and disaccharide **o9** were hydrolysed by xylosidase to Xyl and left untouched by glucosidase, supporting their identity as xylotriose and xylobiose, respectively. The di- and trisaccharides **o8** and **o7** were also hydrolysed by xylosidase, releasing Xyl ≈ Glc and Xyl > Glc, respectively, indicating that they were Xyl-Glc and Xyl-Xyl-Glc. Supporting this, they were unaffected by glucosidase.

#### Cellulase-generated oligosaccharides

The cellulase-derived substrates were not completely pure, and contaminants can be seen in compounds **o10–o14** (Fig. 7D); however, it is still possible to draw conclusions. The disaccharides **o13** and **o14** were hydrolysed by glucosidase and xylosidase, yielding Glc and Xyl ≈ Glc, respectively, hence they are concluded to be Glc-Glc and Xyl-Glc. One of the trisaccharides, **o12**, was hydrolysed only by xylosidase, yielding Xyl > Glc, indicating Xyl-Xyl-Glc. The other trisaccharide, **o11**, was hydrolysed only by glucosidase, yielding Glc, indicating Glc-Glc-Glc. The tetrasaccharide **o10** was hydrolysed by xylosidase, yielding mainly Xyl > Glc, suggesting Xyl-Xyl-Xyl-Glc.

### Alkaline peeling to explore the reducing termini of oligosaccharides

#### Authentic oligosaccharides

All 13 oligosaccharides (**o2–o14**), in addition to cellobiose and xylobiose, were assayed for their lability during alkaline peeling (Fig. 8). This treatment removes sugars progressively from the reducing terminus of an oligosaccharide (Whistler and BeMiller, 1958). In 0.2 M NaOH at 50 °C, the half-lives are <5 min for (1→3) bonds, 20–30 min for (1→4) bonds, and 45–60 min for (1→6) bonds; (1→2) bonds are essentially stable (Fig. S5 of Franková and Fry, 2012), and (1→5) bonds are predicted to resemble (1→6) bonds. The monosaccharide removed from the reducing terminus is released in the form of a saccharinic acid that is not detected by thymol on TLC. Free monosaccharides themselves are more alkali-stable than (1→3), (1→4) and (1→6)-linked oligosaccharides. Therefore, in the case of linear oligosaccharides, after progressive ‘peeling’ from the reducing terminus, the non-reducing terminus can be recovered as a free monosaccharide. Accompanying the ‘peeling’ reaction(s), the reducing terminal sugar may undergo a reversible isomerization (e.g. glucose ↔ fructose ↔ mannose) with no change in DP (Lobry de Bruyn and Alberda van Ekenstein, 1895).

**Fig. 8.**
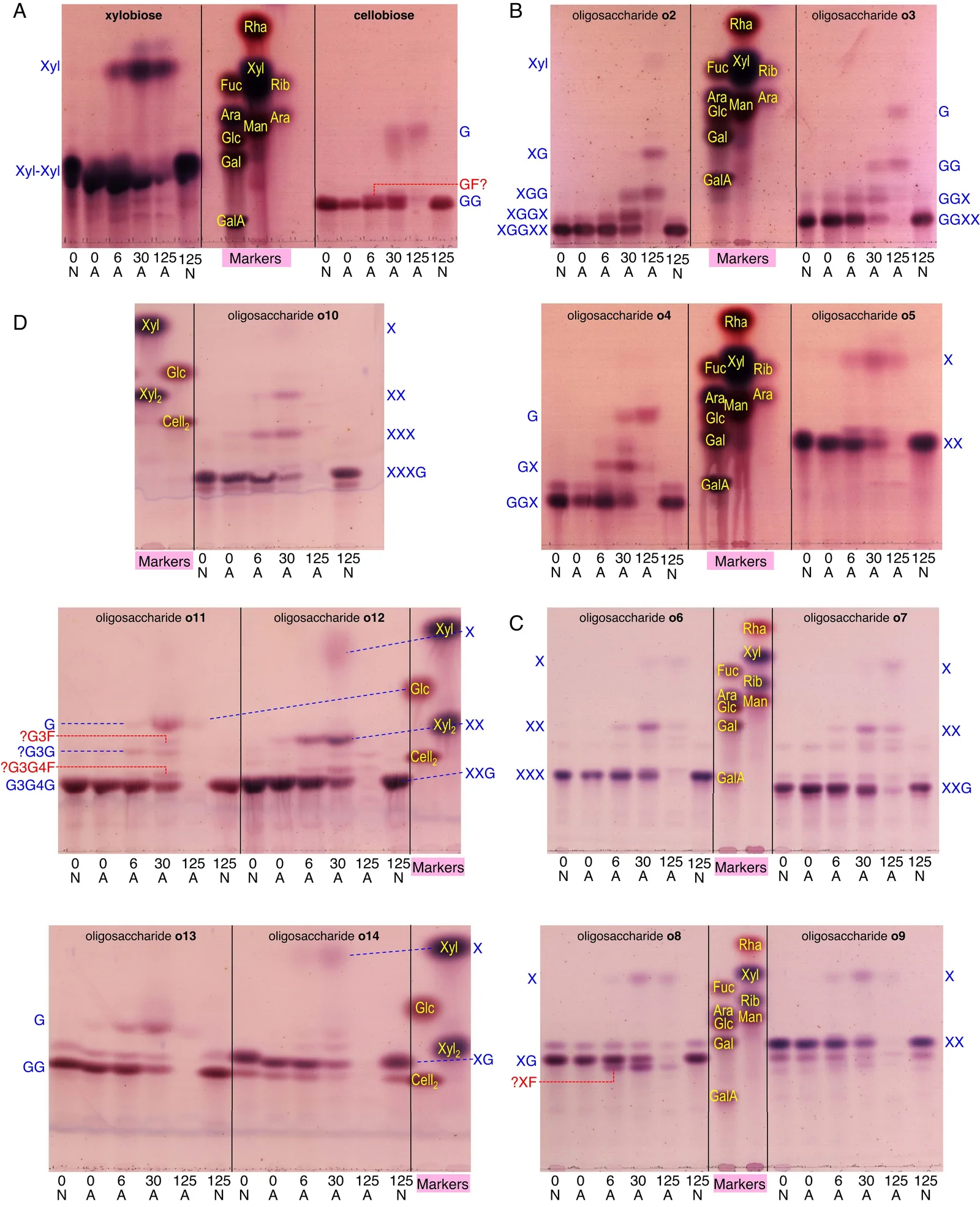
Alkaline peeling of standards and enzymically produced oligomers. Each compound was submitted to alkaline peeling (‘A’ in the loading labels) in 0.2 M NaOH at 50 °C for 0–125 min. Substrates were also incubated at neutral pH (‘N’ in the loading labels) for 125 min as a control. (A) Authentic xylobiose and cellobiose. (B–D) Oligosaccharides were produced from *Ulva* hemicellulose b by xylanase (B), Driselase (C) and cellulase (D). Products were separated by TLC in BAW 2:1:1 with one ascent. Blue labels indicate proposed identities of initial oligosaccharide and products formed by their alkaline peeling. Red labels indicate possible products of isomerization reactions competing with alkaline peeling. F = proposed fructose and/or mannose reducing terminus. Note that abbreviated oligosaccharide names are based on X = xylose and G = glucose; they are not the names used for land-plant xyloglucan oligosaccharides (thus ‘XG’ in this paper implies Xyl-Glc, not Xyl-Glc-Glc).

Both xylobiose and cellobiose have a β-(1→4) glycosidic bond. Thus, as expected, they were both ∼50 % lost after ∼30 min in alkali, although xylobiose was detectably starting to peel after 6 min (Fig. 8A). After 125 min, for both compounds, almost none of the initial oligosaccharide remained, but the monosaccharide (from the original non-reducing terminus) was detected.

#### Xylanase-generated oligosaccharides

Of the xylanase-generated oligosaccharides, the disaccharide **o5** (putative xylobiose) showed detectable peeling within 6 min, exceeding 50 % degradation within 30 min; it released xylose (Fig. 8B), in a pattern resembling that of authentic xylobiose (Fig. 8A). The trisaccharide **o4**, provisionally identified as Glc-Xyl-Xyl, also started to peel after 6 min, producing an oligomer (interpreted as Glc-Xyl) followed by Glc; these observations support the proposed constitution of **o4**, undergoing progressive peeling: Glc-Xyl-Xyl → Glc-Xyl → Glc. The tetrasaccharide **o3** had a similar pattern of peeling, starting after 6 min and gradually continuing in a sequence interpreted as Glc-Glc-Xyl-Xyl → Glc-Glc-Xyl → Glc-Glc → Glc. The pentasaccharide(s) in band **o2** also behaved in a similar manner, the major component apparently peeling in the sequence Xyl-Glc-Glc-Xyl-Xyl → Xyl-Glc-Glc-Xyl → Xyl-Glc-Glc → Xyl-Glc → Xyl.

#### Driselase-generated oligosaccharides

The disaccharide **o9** started to degrade after 6 min and was ∼50 % degraded after 30 min, releasing Xyl, thus resembling xylobiose. Another disaccharide, **o8**, showed a similar pattern of peeling, although slightly slower than **o9**, as expected for Xyl-Glc → Xyl. The trisaccharide **o7** initially left a disaccharide resembling xylobiose, which then itself peeled to release Xyl. Coupled with the observed monosaccharide composition of **o7**, this is interpreted as Xyl-Xyl-Glc → Xyl-Xyl → Xyl. Another trisaccharide, **o6**, followed a similar peeling pattern, showing the same products, interpreted as the sequence Xyl-Xyl-Xyl → Xyl-Xyl → Xyl.

#### Cellulase-generated oligosaccharides

The disaccharide **o14**, composed of Xyl and Glc, was half peeled within ∼30 min, yielding Xyl, indicating that it was Xyl-Glc, not Glc-Xyl. The other disaccharide, **o13**, composed only of Glc residues, was half peeled within ∼30 min, yielding Glc, as expected for Glc-Glc. The trisaccharide **o12** (Glc and Xyl residues) peeled slightly faster, yielding xylobiose, then Xyl, indicating Xyl-Xyl-Glc → Xyl-Xyl → Xyl. The other trisaccharide, **o11**, composed of Glc residues, gave a trace of disaccharide (migrating slightly faster than cellobiose), followed rapidly by a prominent Glc spot. In the light of the NMR data (see below), this behaviour is compatible with Glc-(1→3)-Glc-(1→4)-Glc → Glc-(1→3)-Glc → Glc [where Glc-(1→3)-Glc is laminaribiose], with these two steps being expected to have half-lives of ∼30 and <5 min, respectively (Franková and Fry, 2012). [Exact comparison with markers was not completely conclusive because of some distortion of the solvent migration on this TLC plate.] The tetrasaccharide **o10**, composed of Xyl and Glc residues, peeled relatively rapidly, yielding a trisaccharide, which itself peeled to form xylobiose, then Xyl, a pattern interpreted as Xyl-Xyl-Xyl-Glc → Xyl-Xyl-Xyl → Xyl-Xyl → Xyl.

#### General conclusions from alkaline peeling

The conclusions are summarized in Table 1. During alkaline peeling, each of the 13 *Ulva* oligosaccharides showed a ∼50 % loss within 30 min, compatible with the bond between the penultimate residue and the reducing terminus being (1→4). Moreover, with one exception, each of the subsequent steps (if any) in the sequential peeling pattern of any individual oligosaccharide had a similar half-life, suggesting that all other bonds were also (1→4). The exception was oligosaccharide **o11** (see above). The figure of ‘∼50 %’ within 30 min is a rough approximation, but is adequate to let us state with some confidence that the linkages are not (1→2) or (1→3). The possibility of (1→5) and (1→6) linkages cannot be excluded by this method, but these are likely to be somewhat more stable than (1→4) bonds [demonstrated for (1→6) linkages by Franková and Fry (2012)], and thus not prevalent in our work.

After complete peeling of an oligosaccharide, the free monosaccharide obtained, representing the original non-reducing terminus, persisted to 125 min in most cases; thus, the monosaccharide observed among peeling products indicates the nature of the non-reducing terminus of the oligosaccharide. This was Xyl in the case of **o2**, **o5**, **o6**, **o7**, **o8**, **o9**, **o10**, **o12** and **o14**, and Glc in the case of **o3**, **o4**, **o11** and **o13**, as predicted by the xylosidase and glucosidase conclusions (Fig. 7).

### Conclusion on structures and NMR confirmation

By combining all the data from gel-permeation chromatography, acid hydrolysis, glycosidase digestion and alkaline peeling, we were able to deduce structures for each oligosaccharide (Table 1). These structures were confirmed by the NMR spectra [Supplementary Data Table S1; peak details are omitted for cellobiose (o13) in this table because it is already well characterized in the literature]. The unexpected behaviour of **o11** on alkaline peeling (Fig. 8D: a trisaccharide of Glc peeling to free Glc, with only a very transient intermediary disaccharide) was explained by the NMR data (Table 2), which indicated that this compound, unlike all the others, contained a (1→3) bond in the form Glc-(1→3)-Glc-(1→4)-Glc, i.e. 3-*O*-β-D-glucosyl-cellobiose or G3G4G. This trimer would be expected to peel slowly (half-life ∼30 min) to Glc-(1→3)-Glc (laminaribiose), which would then peel very rapidly (half-life <5 min) to release free Glc.

**Table 2.** NMR spectral data for oligosaccharide **o11** [β-laminaribiosyl-(1→4)-glucose]

Site	δ_C_	δ_H_	Multiplicity	*J*_HH_ (Hz)	HMBC
α-Glc1-1	91.7	5.169	*dd*	*J*_12_ = 3.8	
α-Glc1-2	70.9	3.522	*dd*	*J*_23_ = 10.0	
α-Glc1-3	71.2	3.766	*t*	*J*_34_ = 8.7	
α-Glc1-4	78.3	3.596	*t*	*J*_45_ = 9.4	α-Glc1-C4/Glc2-H1
α-Glc1-5	69.8	3.894	*ddd*	*J*_56a/b_ = 2.5/4.7	α-Glc1-C5/α-Glc1-H1
α-Glc1-6a	59.7	3.834	*dd*	*J*_6a6b_ = 12.4	
α-Glc1-6b		3.806	*dd*		
					
β-Glc1-1	95.6	4.606	*d*	*J*_12_ = 8.0	β-Glc1-C1/β-Glc1-H2
β-Glc1-2	73.8	3.226	*dd*	*J*_23_ = 9.2	
β-Glc1-3	74.0	3.569	*t*	*J*_34_ = 8.9	
β-Glc1-4	78.3	3.607	*t*	*J*_45_ = 9.8	β-Glc1-C4/Glc2-H1
β-Glc1-5	74.8	3.542	*ddd*	*J*_56a/b_ = 2.0/5.0	
β-Glc1-6a	59.6	3.904	*dd*	*J*_6a6b_ = 12.3	
β-Glc1-6b		3.755	*dd*		
					
β-Glc2-1	102.1	4.488	*d*	*J*_12_ = 7.9	Glc2-C1/Glc1-H4(α&β), Glc2-H2
β-Glc2-2	72.9	3.464, 3.471	*2dd*	*J*_23_ = 9.4	
β-Glc2-3	83.6	3.708, 3.711	*2t*	*J*_34_ = 8.3	Glc2-C3/Glc3-H1, Glc2-H2, Glc2-H4
β-Glc2-4	67.7	∼3.46	*ob*	*J*_45_ = *	
β-Glc2-5	75.4	∼3.45	*ob*	*J*_56a/b_ = 2.2/*	
β-Glc2-6a	60.3	3.870	*2dd*	*J*_6a6b_ = 12.4	
β-Glc2-6b		∼3.70	*ob*		
					
β-Glc3-1	102.7	4.697, 4.699	*dd*	*J*_12_ = 7.9	Glc3-C1/Glc2-H3, Glc3-H2
β-Glc3-2	73.3	3.295	*dd*	*J*_23_ = 9.5	
β-Glc3-3	73.4	3.466	*t*	*J*_34_ = 9.4	
β-Glc3-4	69.4	3.350	*t*	*J*_45_ = 9.7	
β-Glc3-5	75.8	3.424	*ddd*	*J*_56a/b_ = 2.2/6.2.0	
β-Glc3-6b	60.5	3.866	*dd*	*J*_6a6b_ = 12.3	
β-Glc3-6a		3.666	*dd*		

^*^Unresolved (signal overlap).

### Study of the affinity of *Ulva* Hb for cellulose

Land-plant hemicelluloses, which are extractable in alkali, are noted for their ability to hydrogen-bond to cellulose (Carpita, 1984; Hayashi, 1989; Edelmann and Fry, 1992). We therefore tested whether the alkali-extracted ‘hemicellulose’ from *Ulva* shares this property. A solution of *Ulva* hemicellulose b was incubated with Whatman No. 1 paper (almost pure cellulose) for various times (Fig. 2). Several land-plant polysaccharides were also included for comparison.

All polymers tested showed some bonding to cellulose: their concentration in solution dropped, immediately after mixing, by 4–20 %. Solutions of the land-plant hemicelluloses interacted only weakly with the cellulose in these conditions: after 72 h, their concentrations had dropped by <20 %, with mixed-linkage glucan (MLG) showing the greatest bonding (∼18–19 %). In comparison, the concentration of *Ulva* hemicellulose b dropped by 44 % within 72 h, which was more than twice as much as any land-plant hemicellulose and which indicates a particularly avid attachment to the filter-paper cellulose.

## DISCUSSION

The alkali-soluble (‘hemicellulose’) fraction from *Ulva* cell walls had previously been characterized only in part. Our work here presents a more detailed structure of this hemicellulose, showing that xylose units are not only interspersed within the cellulose-like linear β-(1→4)-glucan, but also organized as contiguous oligo-Xyl stretches of various lengths (Fig. 2). We therefore propose to name this polysaccharide phycoxyloglucan, to distinguish it clearly from the much better-studied land-plant xyloglucan, whose Xyl residues are α-linked as side-chains to a β-(1→4)-glucan backbone (Fig. 2). The *Ulva* phycoxyloglucan reported here also differs fundamentally from the β-(1→4)-xylan-based ‘pectin’ found in the early-diverging charophyte, *Klebsormidium*. Unlike *Ulva* hemicellulose, the *Klebsormidium* ‘pectin’ is easily solubilized from the cell walls in hot neutral buffers and possesses side-chains of rhamnose and galactose (Rapin *et al.*, 2024).

Although the distribution of oligo-Xyl stretches within the polysaccharide remains unclear, it would be expected to have a direct impact on the properties of the polymer. Specifically, the affinity of the polymer for cellulose could be affected strongly by the presence or absence of these oligo-Xyl segments. The affinity data (Fig. 2) show a remarkable passive affinity of phycoxyloglucan for cellulose, which suggests a similar role in the cell wall, involving hydrogen-bonding to cellulosic microfibrillar surfaces. Such a role has long been proposed for land-plant xyloglucan, but phycoxyloglucan has a considerably greater ability to bond to cellulose from aqueous solution.

The methodology used for characterizing the sequences in phycoxyloglucan was based on ‘enzymic dissection’ using xylanase, Driselase and cellulase (Figs 5 and 6). These enzymes yielded a battery of oligosaccharides of DP 2–5, from which sequences of DP 2–6 could be deduced based on the demonstrated substrate specificity of the three enzyme preparations used (Fig. 4). Analytical methods used for characterizing the oligosaccharides included gel-permeation chromatography to estimate DP, acid hydrolysis to determine the monosaccharide building blocks (Glc and Xyl only), exo-hydrolase digestion to identify the non-reducing terminus, alkaline peeling to confirm the non-reducing terminus (Fig. 8) and to identify the glycosidic bonds present [almost all (1→4)], and NMR spectroscopy to validate further the proposed structures. The conclusion was a series of linear β-(1→4)-linked oligosaccharides that included short oligo-Xyl and oligo-Glc sequences. The one exception was the finding of a Glc-β-(1→3)-Glc unit; this was only observed following cellulase digestion and, based on the demonstrated ability of the ‘cellulase’ to catalyse transglucosylation (Fig. 4), we presume that this is an artefact, in which a small proportion of (1→4) linkages were converted to (1→3) linkages during the cellulase digestion.

The abundance of *Ulva* (visualized in Fig. 1) invites exploration of potential future biotechnological applications for its phycoxyloglucan, which constitutes ∼9 % of the AIR dry weight. In the future, it will be interesting to study the impact of external conditions (e.g. temperature of the water, salinity), which can drastically affect the morphology of *Ulva* (Supplementary Data Fig. S5), on the presence and the distribution of the hitherto unreported oligo-Xyl runs in phycoxyloglucan. This could have a cascading impact on the affinity of the polysaccharide for cellulose, leading to potential changes in the mechanical properties of the algal cell wall and suggesting diverse biotechnological applications for this algal hemicellulose.

Starting from the structural analysis performed in this work, another research axis springs up that is centred around the evolutionary features of alkali-extractable polymers. Characterizing these in different *Ulva* species could be a first step towards testing whether the polysaccharide structure is present within the whole genus, before expanding this search throughout the order Ulvales. Studying the biosynthetic pathway for this polymer, in addition to the genes involved, would provide important information regarding the evolution of phycoxyloglucan and, in particular, help to shed light on whether its evolutionary trajectory is linked to that of the fibrillar algal xylans or to land-plant xylans.

### Conclusions

The chlorophytic seaweed *Ulva* cf. *linza* possesses cellulose, ‘pectin-like’ anionic polysaccharides and a hitherto poorly characterized hemicellulose that can be extracted in concentrated alkali. Here, we show that *Ulva* hemicellulose has an unbranched backbone of β-Xyl and β-Glc residues. In this sense it is a ‘xyloglucan’; however, it differs fundamentally from land-plant xyloglucans in having β-Xyl (not only β-Glc) residues in the backbone and in lacking α-Xyl side-chains, and we therefore propose to term it phycoxyloglucan. It might have been speculated that *Ulva* cell-wall polysaccharides possibly resemble those of their closest streptophyte relatives, such as the early-diverging charophytes *Chlorokybus* and *Klebsormidium*. However, *Ulva* phycoxyloglucan also differs fundamentally from the β-xylan-based polysaccharides of *Klebsormidium*, which have rhamnose and galactose side-chains and are extractable from the cell wall in hot, near-neutral water. Unusually, in the light of previously studied hemicelluloses, we show that the backbone of phycoxyloglucan includes runs of up to three contiguous Xyl residues and up to three contiguous Glc residues, giving a structure that appears to hydrogen-bond to cellulose more rapidly than land-plant xyloglucan and other tested land-plant polysaccharides. This avid hemicellulose–cellulose bonding might enhance the mechanical properties of the *Ulva* cell wall and suggests new biotechnological applications for the polysaccharides of this seaweed.

## Supplementary Material

mcag195_Supplementary_Data

## SUPPLEMENTARY DATA

Supplementary data are available at *Annals of Botany* online and consist of the following. Table S1: NMR spectra of *Ulva* oligosaccharides. Figure S1: gel-permeation and preparative paper chromatography to isolate oligosaccharide fragments of *Ulva* hemicellulose. Figure S2: separation of *Ulva* oligosaccharide **o6** from **o7** and **o11** from **o12**. Figure S3: acid hydrolysis products of *Ulva* hemicellulose oligosaccharides. Figure S4: co-migration of *Ulva* oligosaccharides o5, o9 and o13 with authentic xylobiose or cellobiose. Figure S5: *Ulva* cf. *linza*, showing two morphologies.
