# Supplementary material for: Linear phycoxyloglucan with oligo-xylose domains in the green seaweed Ulva: a unique hemicellulose
Source: Ann Bot. 2026 Jul 17;138(5):1738–52. doi: 10.1093/aob/mcag195 (PMC13715020; doi:10.1093/aob/mcag195)
Supplement: Supplementary file 1 [file mcag195_supplementary_data.zip › Table S1.docx]

**Table S1**

Table S1 cont’d

Table S1 cont’d

Table S1 cont’d

Table S1 cont’d

Table S1 cont’d

Table S1 cont’d

Table S1 cont’d

Table S1 cont’d

Table S1 cont’d

Table S1 cont’d
